# Concepts and considerations for enhancing RNAi efficiency in phytopathogenic fungi for RNAi-based crop protection using nanocarrier-mediated dsRNA delivery systems

**DOI:** 10.3389/ffunb.2022.977502

**Published:** 2022-09-08

**Authors:** Poonam Ray, Debashish Sahu, Raghavendra Aminedi, Divya Chandran

**Affiliations:** ^1^ Laboratory of Plant-Microbe Interactions, Regional Centre for Biotechnology, NCR Biotech Science Cluster, Faridabad, India; ^2^ Division of Genomic Resources, ICAR-National Bureau of Plant Genetic Resources, New Delhi, India

**Keywords:** gene silencing, siRNA/dsRNA, HIGS, SIGS, off-target effects, nanoparticles, LDH, chitosan

## Abstract

Existing, emerging, and reemerging strains of phytopathogenic fungi pose a significant threat to agricultural productivity globally. This risk is further exacerbated by the lack of resistance source(s) in plants or a breakdown of resistance by pathogens through co-evolution. In recent years, attenuation of essential pathogen gene(s) *via* double-stranded (ds) RNA-mediated RNA interference (RNAi) in host plants, a phenomenon known as host-induced gene silencing, has gained significant attention as a way to combat pathogen attack. Yet, due to biosafety concerns regarding transgenics, country-specific GMO legislation has limited the practical application of desirable attributes in plants. The topical application of dsRNA/siRNA targeting essential fungal gene(s) through spray-induced gene silencing (SIGS) on host plants has opened up a transgene-free avenue for crop protection. However, several factors influence the outcome of RNAi, including but not limited to RNAi mechanism in plant/fungi, dsRNA/siRNA uptake efficiency, dsRNA/siRNA design parameters, dsRNA stability and delivery strategy, off-target effects, etc. This review emphasizes the significance of these factors and suggests appropriate measures to consider while designing *in silico* and *in vitro* experiments for successful RNAi in open-field conditions. We also highlight prospective nanoparticles as smart delivery vehicles for deploying RNAi molecules in plant systems for long-term crop protection and ecosystem compatibility. Lastly, we provide specific directions for future investigations that focus on blending nanotechnology and RNAi-based fungal control for practical applications.

## 1 Introduction

Fungal diseases have long been recognized as a pervasive threat to agricultural productivity and global food security (Fones et al., 2020; Ristaino et al., 2021), causing enormous economic losses worldwide. Phytopathogenic fungi are typically more prone to epidemics owing to their high virulence, broad host range, and ability to persist under adverse environmental conditions and evolve new lineages (Fisher et al., 2012). Modeling studies predict that the rise in global temperatures, trade, and transport may exacerbate this problem and facilitate the emergence of new fungal diseases, particularly in fragile ecological terrains (Yadav et al., 2020; Ristaino et al., 2021). A major fungal epidemic can also indirectly affect food systems in underdeveloped countries where restrictive global trade policies may trigger a substantial hike in crop prices (Godfray et al., 2016).

Current fungal disease management practices include the use of chemical/bio-fungicides and the cultivation of genetically resistant plant varieties. Although fungal control through chemical fungicides has been largely successful, the efficacy of the treatment is influenced by the type of fungicide, the timing and the number of applications, and abiotic factors (Bandara et al., 2020; Carmona et al., 2020). Some fungicides negatively impact non-target beneficial microorganisms (Yang et al., 2011; Karlsson et al., 2014) and human health (Pearson et al., 2016), and may also exhibit phytotoxicity, adversely affecting photosynthesis and plant biomass production (Dias, 2012). The excessive use of fungicides also results in the development of resistant fungal strains (Vielba-Fernández et al., 2020). Bio-control agents are eco-friendly alternatives to chemical fungicides (El-Baky and Amara, 2021; Leannec-Rialland et al., 2022; Tyśkiewicz et al., 2022), but their effectiveness is heavily dependent on environmental factors (Ons et al., 2020). Breeding genetically-resistant plants is currently the most popular and sustainable strategy for fungal disease management. Nevertheless, natural sources of resistance, particularly against new pathogen races, are challenging to develop during a fungal outbreak.

**Figure 1:**
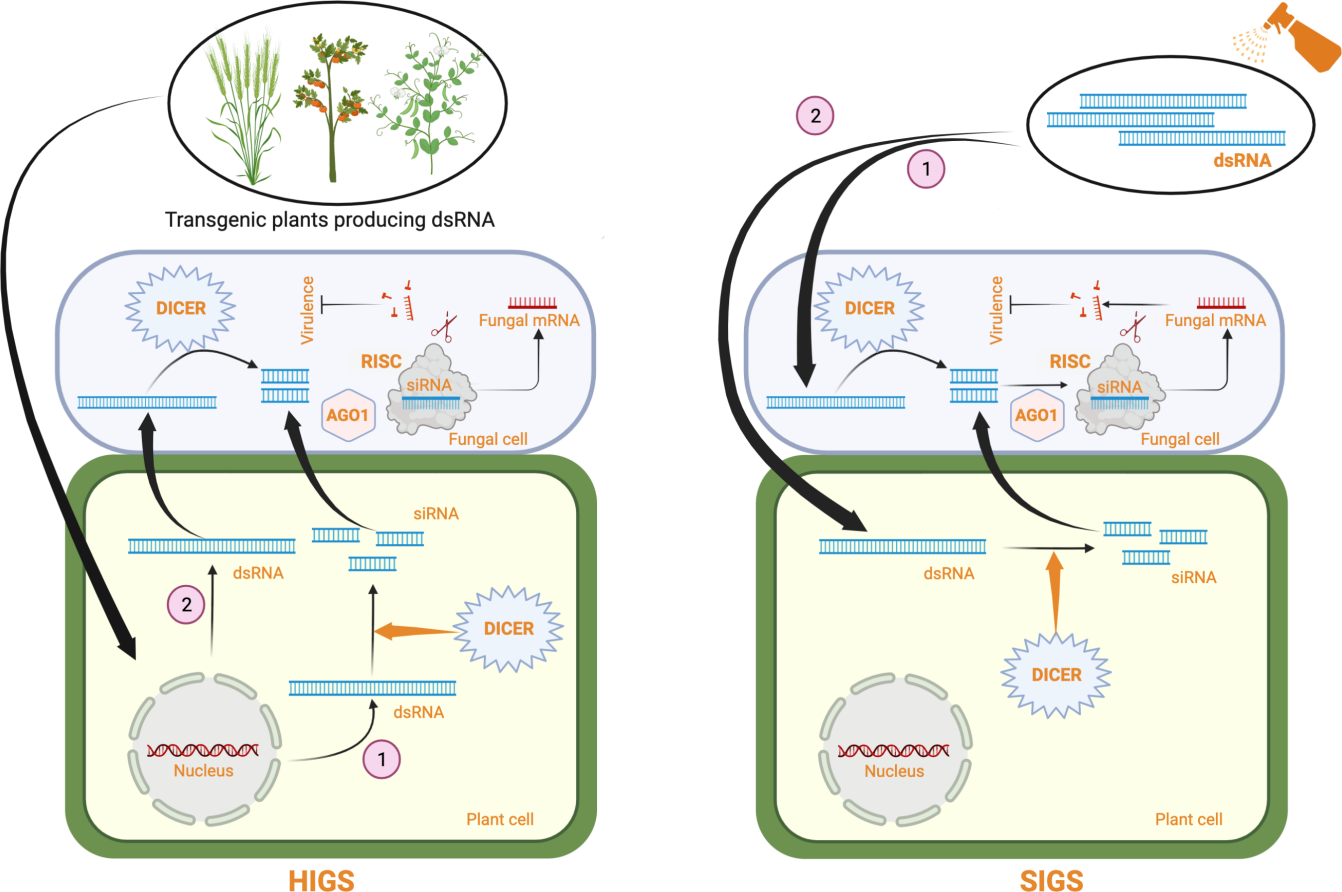
A schematic representing RNAi-based fungal control strategies in plants using dsRNA. In HIGS, a dsRNA producing construct specific for an essential fungal gene is stably or transiently (not shown) expressed in plants. Some dsRNAs are processed by plant DICER proteins producing siRNAs (1) which are transferred to the fungal cell. Some dsRNAs are directly taken up by the fungal cell (2) and cleaved by pathogen DICER proteins. siRNAs produced are incorporated into the RISC complex through interaction with fungal argonaute (AGO1) proteins. The siRNA containing RISC complexes bind to the endogenous fungal target mRNAs based on sequence complementarity and degrade them, thus inhibiting pathogen growth and virulence. In SIGS, naked dsRNAs are directly sprayed on to the plant surface. The dsRNAs can directly go into the fungal cell where they are cleaved by fungal DICER proteins into siRNAs (1) or the dsRNAs can enter the plant cell where they are processed by plant DICER proteins into siRNAs and the resulting siRNAs are taken up by the fungal cell. The siRNAs degrade endogenous fungal target mRNAs through the same mechanism as described for HIGS, inhibiting fungal growth and virulence. Created with BioRender.com.

RNA interference (RNAi)-mediated gene silencing has emerged as a powerful tool for crop protection against various biotic stresses, including fungal pathogens (Zotti et al., 2018; Kaur et al., 2021). This method, also known as host-induced gene silencing (HIGS), exploits the intrinsic ability of the plant RNAi machinery to process fungal-specific long double-stranded RNAs (dsRNAs) into short-interfering RNAs (siRNAs), which the pathogen can subsequently internalize for sequence complementarity-based silencing of fungal genes (reviewed in Nunes and Dean, 2012; Qi et al., 2019). In HIGS, hairpin constructs encoding fungal-specific genes are either stably or transiently expressed within plant cells to generate fungal-specific dsRNA for gene silencing (**Figure 1**). HIGS has been successfully used to confer RNAi-mediated resistance against several fungal pathogens, including *Blumeria graminis*, *Puccinia spp*, *Fusarium spp*, *Verticillium dahlia*, *Magnaporthe oryzae*, and *Aspergillus flavus* (summarized in **Table 1**). However, using transgenic approaches for the stable expression of fungal-specific dsRNAs in plant cells is often technically challenging and time-consuming. These factors and the limited public acceptance of genetically modified (GM) crops have restricted the adoption of HIGS in large-scale applications. Therefore, a transgene-free method known as spray-induced gene silencing (SIGS) was established, in which fungal-specific dsRNAs or siRNAs are directly applied to plant tissues for targeted silencing of fungal genes [**Figure 1** **;** (Wang et al., 2016; Koch et al., 2016; Cai et al., 2018) and comprehensively reviewed in (Vetukuri et al., 2021)].

**Table 1:** List of phytopathogenic fungal genes targeted through Host-Induced Gene Silencing (HIGS).

Host	Fungal pathogen	Target gene(s)^1^	Role of Target gene(s)	Silencing outcome	Reference
Maize	*Aspergillus flavus*	*Amy1*	Role in early pathogen infection	Reduced fungal growth & aflatoxin accumulation	Gilbert et al., 2018
Maize	*A. flavus*	*Alk*	Virulence	84% reduction in aflatoxin accumulation and reduced fungal biomass	Omolehin et al., 2021
Wheat	*Blumeria graminis f.* sp. *tritici*	*MLO*	Susceptibility factor	Resistance	Riechen, 2007
Barley, Wheat	*B. graminis*	*Avra10*	Avirulence effector	Reduced fungal development in the absence of *Mla10 R* gene	Nowara et al., 2010
Wheat, Barley	*B. graminis*	*BEC (1011, 1054, 1038, 1016, 1005, 1019, 1040*, *1018)*	Ribonuclease-like effectors	Reduced virulence	Pliego et al., 2013
Potato, Tomato	*Botrytis cinerea*	*TOR*	Regulation of growth and metabolism	Significant reduction of grey mould	Xiong et al., 2019
Tobacco	*Ciboria shiraiana*	*Gpa1*	Virulence; appressorium formation	Fewer necrotic lesions and reduced pathogen biomass	Zhu et al., 2021
Tomato, Chili	*Colletotrichum gloeosporioides*	*COM1*	Fungal conidial and appressorium formation	Suppression of conidial germination, germ tube development, appressoria formation and mycelial growth	Mahto et al., 2020
Barley	*Fusarium graminearum*	*CYP51A, CYP51B, CYP51C*	Ergosterol biosynthesis	Complete reduction of fungal growth with no disease symptoms	Koch et al., 2013
Banana	*F. oxysporum f.* sp. *cubense*	*VEL, FTF1*	Regulators of (a)sexual development, secondary metabolism and virulence	7 to 25-fold reduction in conidiophore count	Ghag et al., 2014
Wheat	*F. graminearum*	*Chs3b*	Chitin synthesis	21-47% reduction in lesion length; 63-73% reduction in infected spikelets	Cheng et al., 2015
Arabidopsis	*F. oxysporum*	*FRP1*, *FOW2*, *OPR*	Pathogenicity (*FRP1, FOW2*)*;* Jasmonic acid biosynthesis (*OPR*)	25% in survival rate of *FOW2*-silenced lines, 30–50% in *FRP1*-silenced lines, and 45-70% in *OPR*-silenced lines	Hu et al., 2016
Wheat	*F. culmorum*	*Gls1*	Chitin synthesis	50–60% reduction in disease symptoms	Chen et al., 2016
Tomato	*F. oxysporum f.* sp. *lycopersici*	*FOW2, ChsV*	Putative transcription regulator controlling infection; chitin synthesis	Enhanced disease resistance	Bharti et al., 2017
Wheat	*F. graminearum*	*SGE1, PP1, STE12*	Deoxynivalenol (DON) biosynthesis regulator (*SGE1)*; Transcriptional regulator of penetration structure formation *(STE12)*	Reduction in fungal infection structures, inhibition of DON biosynthesis, and enhanced resistance	Wang et al., 2020
Banana	*F. oxysporum* TR4	*ERG6, ERG11*	Ergosterol biosynthesis	Enhanced disease resistance	Dou et al., 2020
Soybean	*F. oxysporum*	*Chs*	Chitin synthesis	Reduction in lesion size & fungal biomass	Kong et al., 2022
Rice	*Magnaporthe oryzae*	*ABC, MAC1, PMK1*	Pathogenicity factors	Enhanced disease resistance	Zhu et al., 2017a
Rice	*M. oryzae*	*AP1, SSADH, ACT, SOM1*	Transcription factors	Only *AP1* silencing led to reduced aerial hyphal growth & enhanced resistance	Guo et al., 2019
Barley, Wheat	*Puccinia striiformis f.* sp. *tritici, P. graminis f.* sp. *tritici*	*Pstha12J12*	Encodes secreted protein	No obvious reduction in rust development or sporulation	Yin et al., 2011
Wheat	*P. triticina, P. graminis, P. striiformis*	*PtMAPK1*, *PtCYC1*, *PtCNB*	Pathogenicity factors	Disease suppression, compromises fungal growth and sporulation	Panwar et al., 2013
Wheat	*P. striiformis f.* sp. *tritici*	*CPK1*	involved in virulence	Significant reduction in the infection hyphae length and disease phenotype	Qi et al., 2018
Wheat	*P. striiformis f.* sp. *tritici*	*Fuz7*	MAPK kinase, regulates pathogen infection and development	Restricted hyphal development and induction of necrosis in plant cells	Zhu et al., 2017b
Wheat	*P. triticina*	*MAPK1, CYC1*	Pathogenicity factors	Reduced fungal biomass accumulation	Panwar et al., 2018
Tall fescue	*Rhizoctonia solani*	*RNA polymerase, Imbs, Coh, UbiE3*	Essential for pathogen growth	90% reduction in disease lesion size	Zhou et al., 2016
Rice	*R. solani*	*tps2*	Trehalose biosynthesis	Reduced trehalose accumulation & virulence	Zhao et al., 2021
Arabidopsis	*Sclerotinia sclerotiorum*	*oah1*	Negative regulator of plant defense	Decreased accumulation of oxalic acid in necrotic regions & reduced pathogenesis	Rana et al., 2022
Rice	*Ustilaginoidea virens*	*Chs2*, *Chs5*	Chitin synthesis	Reduction in disease symptoms	Li et al., 2021
Rice	*U. virens*	*COM1, Pro1, AspE*	Virulence	Inhibition of hyphae extension and enhanced disease resistance	Chen et al., 2022
Cotton	*Verticillium dahliae*	*VdH1*	Melanized microsclerotia formation	50–75% reduction in disease symptoms	Zhang et al., 2016
Arabidopsis	*V. dahliae*	*Ave1, Sge1, NLP1*	Pathogenicity factors	Reduced disease symptoms	Song and Thomma, 2018
Cotton	*V. dahliae*	*RGS1*	Regulator of G protein signaling involved in spore production, hyphal development, microsclerotia formation	Broad spectrum resistance to *V. dahliae* strains	Xu et al., 2018
Cotton	*V. dahliae*	*ALS* subunits *ILV2, ILV6*	Branched-chain amino acid synthesis	Drastic reduction in disease development	Wei et al., 2020

^1^MLO, Mildew-Locus O; Pstha12J12, haustorial transcript; PtMAPK1, mitogen activated protein kinase, PtCYC1, cyclophilin; PtCNB, calcineurin B; CYP, cytochrome P450; BEC, Blumeria effector candidates; VEL, velvet; FTF1, Fusarium transcription factor; Chs, Chitin synthase; FRP1, F-box protein required for Pathogenicity 1; FOW2, F. oxysporum Wilt 2; OPR, 12-oxophytodienoate-10,11-reductase; FcGls1, ß-1, 3-glucan synthase; VdH1, Hygrophobins1; Imbs, Importin ß-1 subunit; Coh, Cohesin complex subunit; UbiE3, Ubiquitin E3 ligase; PsCPK1, calcium dependent protein kinase 1; VdRGS1, regulators of G protein signaling; TOR, Target of rapamycin; ALS, Acetolactate synthase; COM1, conidium morphology mutant 1; FgSGE1, SIX Gene Expression 1; FgPP1, protein phosphatases; ERG, ergosterol; Rstps, trehalose-6-phosphate phosphatase; CsGpa1, G protein alfa subunit; Alk, alkaline protease; Ssoah1, oxaloacetate acetylhydrolase; Pro1, protoperithecia forming mutant.

Unlike chemical fungicide-based control strategies, RNAi-based crop protection methods (HIGS and SIGS) offer eco-friendly methods to combat plant fungal pathogens. The high specificity of the RNAi mechanism, its presence in nearly all eukaryotes, and the regular consumption of RNA by humans in the form of fresh fruits and vegetables suggest that RNAi-based disease control is likely to be non-toxic (Fletcher et al., 2020). Neither HIGS nor SIGS involves the expression of foreign proteins, further reducing the toxic side effects following ingestion by animals/humans. However, SIGS offers several advantages over the HIGS approach. Unlike HIGS, SIGS does not mandate the development of efficient transformation method(s) for each crop species, nor does it confine the technology to a specific gene or application (Taning et al., 2020). As a result, the exogenous method has the potential to be more readily accepted by the public and biosafety regulators. It is also faster to optimize than the generation of a stable HIGS plant. In addition, topically administered dsRNA may not pose environmental risks as dsRNAs are rapidly degraded (half-life less than 30-72 h) in soil and water (Dubelman et al., 2014; Fischer et al., 2017; Zhang et al., 2020). The biodegradability of RNA, however, creates a significant bottleneck for RNAi application in crops as it offers a narrow protection window of only a few days (Landry and Mitter, 2019). For effective and long-term RNAi, dsRNA molecules must be protected from environmental degradation and released into plant cells in a controlled manner. Recent research efforts have focused on nanoparticles as smart delivery vehicles of RNAi molecules to enhance the stability of dsRNAs and permit their controlled release into plant cells, providing longer disease protection windows (Mujtaba et al., 2021; Avila-Quezada et al., 2022).

This review summarizes the studies that have used SIGS for RNAi in phytopathogenic fungi and emphasizes the critical parameters to be considered for designing effective SIGS-based RNAi under lab and field conditions. It also introduces nanoparticles as promising carriers of RNA molecules and presents a set of proven and probable nanocarriers that can enhance the efficiency of dsRNA delivery into plant cells for long-term RNAi-based protection against fungal diseases in crops.

## 2 Spray-induced gene silencing (SIGS)

Spray-induced gene silencing or SIGS is a technique that involves spraying dsRNA onto the plant surface for targeted RNAi. In SIGS, the exogenously applied dsRNA can either be directly taken up by the fungal cells (environmental RNAi) (Wang et al., 2016; Koch et al., 2016; McLoughlin et al., 2018; Qiao et al., 2021) or transit through plant cells (cross-kingdom RNAi) (Wang et al., 2016; Cai et al., 2018), in contrast to HIGS, where the dsRNA is stably/transiently expressed in plant cells, and the RNAi molecules (dsRNA/siRNA) are delivered to the fungal cells *via* plant cells [(**Figure 1**); (Nunes and Dean, 2012)]. The RNAi molecules that enter the fungal cells are processed by the fungal RNAi machinery for targeted gene silencing, resulting in fungal growth arrest (Dang et al., 2011). The first patented study on exogenous delivery of *in vitro*-transcribed dsRNAs (685 bp) and chemically synthesized 21-mer antisense ssDNA molecules on plants, triggering RNAi of the plant endogenous gene *phytoene desaturase*, was reported in *Nicotiana benthamiana* (Sammons et al., 2018). Since then, several studies have demonstrated the ability of exogenously applied dsRNAs/siRNAs to protect crops/model plants against various phytopathogenic fungi, including *Fusarium spp*, *Botrytis cinerea*, *Sclerotinia sclerotiorum*, *Phakopsora* pachyrhizi, *Podosphaera xanthii*, *Verticillium dahliae*, *Colletotrichum spp, Aspergillus niger*, and *Rhizoctonia solani* (summarized in **Table 2**). Therefore, SIGS holds immense potential as a crop protection method against phytopathogenic fungi.

**Table 2:** List of phytopathogenic fungal genes targeted through Spray-Induced Gene Silencing (SIGS).

Host	Fungal pathogen	Target gene(s)	Role of Target gene(s)	Silencing outcome	Reference
Tomato Apple Grape	*Aspergillus niger*	*VPS51+DCTN1+SAC1, pgxB*	Vesicle trafficking; virulence factor	Reduction in *VPS51* (44%)*, DCTN1* (32%)*, SAC1* (75%), and *pgxB* (62%) expression; Reduction in disease symptoms and lesion size	Qiao et al., 2021
Tomato Lettuce Onion Grape Strawberry Rose Arabidopsis	*Botrytis cinerea*	*DCL1, DCL2, DCL1+2*	RNAi process	Reduction in the lesion size and complete inhibition of fungal growth with DCL1+DCL2 constructs	Wang et al., 2016
Canola	*B. cinerea*	peroxidase, TIM44, Thioredoxin reductase, pre-40S ribosomal particle, Necrosis-inducing peptide 1	peroxidase, ROS response, translation, oxidoreductase, mitochondrial translocase	Reduction in lesion size (38-66%) and reduced disease symptoms	McLoughlin et al., 2018
Cucumber	*B. cinerea*	*ß2 tubulin*	Fungal growth	Inhibition of spore germination and mycelia growth; 78% reduction in fungal biomass	Gu et al., 2019
Grape	*B. cinerea*	*BcEF2, BcCYP51, Bcchs1*	Elongation factor, ergosterol and chitinase biosynthesis	Reduction in disease symptoms depending on the application method	Nerva et al., 2020
Tomato Lettuce Grape Rose	*B. cinerea*	*VPS51+DCTN1+SAC1*, *DCL1+DCL2*	Vesicle trafficking; RNAi process	Reduction in *VPS51 and DCTN1* (42%) and *SAC1* (82%) expression; Reduction in disease symptoms and lesion size	Qiao et al., 2021
Soybean	*Colletotrichum truncatum*	*ß2 tubulin*	Fungal growth	Inhibition of spore germination and mycelia growth; 54% reduction in fungal biomass	Gu et al., 2019
Tomato Apple Cherry	*C. gloeosporioides*	*DCL 1 + 2, VPS51+DCTN1+SAC1*	Vesicle trafficking; RNAi process	No uptake of dsRNA resulting in no transcript suppression; disease symptoms indistinguishable from control	Qiao et al., 2021
Wheat	*F. asiaticum*	*Myosin 5*	Cytokinesis and actin filaments organization	Reduced pathogenicity & fungicide resistance	Song et al., 2018
Barley	*F. graminearum*	*CYP51*	Ergosterol biosynthesis	80% reduction in infection; 50% reduction in expression	Koch et al., 2019
Wheat	*F. asiaticum*	*ß2 tubulin*	Fungal growth	Formation of crooked and exiguous mycelia and 90% reduction in fungal biomass, reduction in resistance to carbendazim	Gu et al., 2019
Wheat	*F. graminearum*	*RdRP1, AGO1, QDE3, QIP, AGO2, DCL1, RdRP2, RdRP3, RdRP4, and DCL2*	RNAi process	Reduced fungal germination, growth & pathogenicity	Gaffar et al., 2019
Barley	*F. graminearum*	*AGO, DCL*	RNAi process	Reduction in *AGO* (40%) and *DCL* (74%) expression; 60% reduction in disease symptoms	Werner et al., 2020
Arabidopsis	*F. graminearum*	*CYP51A, CYP51B, CYP51C*	Ergosterol biosynthesis	Reduction in pathogen growth (52%-82%) depending on the length of the respective dsRNA	Höfle et al., 2020
Soybean	*Phakopsora pachyrhizi*	*Acetyl-CoA acyltransferase, 40S ribosomal protein S16, Glycine cleavage system H protein*	Infection and pathogenecity	~73% reduction of pustule numbers and 75% reduction in fungal biomass accumulation	Hu et al., 2020
Wheat	*F. graminearum*	*Chs7, Gls and Pkc*	Chitin synthesis; Mycelium growth and pathogenicity	63-85% reduction in expression; 43-54% reduction in infected spikelets	Yang et al., 2021
Wheat	*Fusarium culmorum*	*TRI5*	Trichothecene biosynthesis	76% reduction in expression; 5-fold reduction in fungal biomass	Tretiakova et al., 2022
Arabidopsis (*in vitro*)	*Macrophomina phaseolina*	*CHS6*	Chitin synthase	Reduction in the fungal growth (30-40%), and fungal biomass (60-70%)	Forster and Shuai, 2020
Barley	*Magnaporthe oryzae*	*ß2 tubulin*	Fungal growth	Inhibition of spore germination and mycelia growth; 80% reduction in fungal biomass	Gu et al., 2019
Rice	*M. oryzae*	*DES1*	Negative regulator of host defense	Reduction in disease symptoms (25-60%); depends on the exposure of host to dsRNA prior to infection	Sarkar and Roy-Barman, 2021
Melon	*Podosphaera xanthii*	*CNAP1048, CNAP10905, CNAP30520*	N-linked glycosylation pathway, respiration	50% reduction in expression; disease severity reduced by 80-90%	Ruiz-Jiménez et al., 2021
Rice	*Rhizoctonia solani*	*DCTN1+SAC1, PG*	Vesicle trafficking; pectin degradation	Reduction in *DCTN1* and *SAC1* (72%) and *PG* (62%) expression; Reduction in disease symptoms and fungal biomass (58% with *DCTN1+SAC1;* 43% with PG)	Qiao et al., 2021
Canola Arabidopsis	*Sclerotinia sclerotiorum*	59 target genes 16 target genes	Cell wall modification, mitochondria, ROS response, protein modification, pathogenicity factors, transcription, splicing, & translation	Reduction in lesion size on Canola (26-85%) and Arabidopsis (34-66%) and reduced disease symptoms	McLoughlin et al., 2018
Lettuce Collard green	*S. sclerotiorum*	*VPS51+DCTN1+SAC1*, *DCL1+DCL2*	Vesicle trafficking; RNAi process	Reduction in *VPS51* (90%), *DCTN1* (78%), & *SAC1* (76%) expression. Reduction in disease symptoms and lesion size	Qiao et al., 2021
Arabidopsis	*Verticillium dahliae*	*DCL 1 + 2*, *DCTN1+ SAC1*	Vesicle trafficking; RNAi process	Reduction in *DCL1* (55%), *DCL2* (62%), *DCTN1* (48%)*, SAC1* (60%) expression. Reduction in disease symptoms & fungal biomass (55% with *DCL1+DCL2;* 60% with *DCTN1+SAC1*)	Qiao et al., 2021

CYP, cytochrome P450; DCL, dicer-like; RdRP, RNA-dependent RNA polymerase; AGO, argonaute; QDE, quelling-defective; QIP, QDE-2-interacting protein; CHS, chitin synthase; EF, elongation factor; DCTN, dynactin; SAC, suppressor of actin; PG, polygalacturonase; VPS, vacuolar protein sorting; pgxB, exo-polygalacturonase b; DES, sphingolipid delta (4)-desaturase; CNAP, conserved and non-annotated protein; Gls, glucan synthase; Pkc, protein kinase C; TRI, trichodiene synthase.

However, numerous factors, including but not limited to target gene selection, dsRNA uptake mechanisms, barriers to dsRNA uptake, dsRNA/siRNA design parameters, dsRNA dose and size, application method, delivery strategy, and environmental stability, may affect the efficacy of exogenously applied RNA molecules to trigger successful RNAi in plant-fungal interactions (Bennett et al., 2020; Das and Sherif, 2020; Šečić and Kogel, 2021; Niu et al., 2021; Hoang et al., 2022). The following sections highlight the importance of each of these factors based on knowledge applicable to fungi and/or other organisms and provide a design manual to fully utilize SIGS as effectively as possible for practical applications (**Table 3**) as well as to promote SIGS as one of the potential anti-fungal protection technologies of today’s generation.

**Table 3:** List of factors influencing dsRNA-mediated RNAi in phytopathogenic fungi.

Parameter	Putative underlying factor in fungi, plant or environment	Fungi investigated	References
Target gene	Essential for normal growth and development	Over 150 target genes investigated on 25 phytopathogenic fungi infecting 22 different hosts	Gebremichael et al., 2021
	Involved in virulence	*Erysiphe pisi* effectors *CSEP001/009;* virulence factor *CSP083*	Sharma et al., 2019
Functional RNAi machinery in fungus	Functional RNAi machinery	*Verticillium nonalfalfae*	Jeseničnik et al., 2019
	Non-functional RNAi machinery	*Zymoseptoria tritici*	Kettles et al., 2019
	Loss of RNAi machinery genes	*Ustilago maydis*	Laurie et al., 2008
Secondary amplification of siRNA	Non-functional RdRP or transiently functional	*Fusarium asiaticum*	Song et al., 2018
Presence of systemic RNAi	Existence of cell-to-cell movement of dsRNA	*F. graminearum*	Koch et al., 2016
dsRNA uptake efficiency in fungus	dsRNA uptake by clathrin-mediated endocytosis (CME)	*Sclerotinia sclerotiorum*	Wytinck et al., 2020
		*Botrytis cinerea*	Wang et al., 2016
	dsRNA uptake by endocytic fusion of extracellular vesicles with plasma membrane	*B. cinerea*	Cai et al., 2018
	Efficient dsRNA uptake	*B. cinerea, S. sclerotiorum, Rhizoctonia solani, Aspergillus niger, V. dahliae*	Qiao et al., 2021
	Limited dsRNA uptake	*Trichoderma virens*	
	No dsRNA uptake	*Colletotrichum gloeosporioides*	
dsRNA stability/degradation	Ability to pass through plant barriers and digestion by plant-generated nucleases	–	Bennett et al., 2020
	Environmental factors	–	Fischer et al., 2016 Parker et al., 2019
dsRNA design -dsRNA/siRNA -dose -length -homology with target -no. of targeted loci	-Amenable to effective RNAi -dsRNA size limitation -Half-life of dsRNA/siRNA -Target expression level	*B. cinerea, S. sclerotiorum, E. pisi, Macrophomina phaseolina, F. graminearum*	McLoughlin et al., 2018 Sharma et al., 2019 Forster and Shuai, 2020 Höfle et al., 2020
Concomitant delivery of dsRNA targeting nuclease	Inactivation of nucleases	–	–
Physiological status of the fungus	-Growth stage -Cell/tissue type	*E. pisi*	Sharma et al., 2019
dsRNA delivery -HIGS -SIGS -Foliar infiltration -Nanoparticles	-Nature of pathogen colonization -Internalization into plant cells -Protection from dsRNA degradation in environment, plant/fungus	Refer **Tables 1**, **2** and **4** (HIGS, SIGS and nanoparticles)	
Off-target effects	-non-target genes -non-target beneficial organisms	*in silico* analyses in *E. pisi*	Sharma et al., 2019

## 3 Factors to consider for effective RNAi in phytopathogenic fungi

### 3.1 Selection of target gene(s)

#### 3.1.1 Target gene function

The success of RNAi-based fungal control critically relies on selecting target genes indispensable for fungal survival. It is generally known that RNAi effectiveness differs significantly in insects between the same transcripts of various species, between genotypes and tissues of the same species, as well as between different regions of the same transcript [reviewed by (Silver et al., 2021)]. Such detailed examination is lacking in plant pathogenic fungi. Various gene candidates have been tested over the last decade to determine whether RNAi works against phytopathogenic fungi (**Table 1**). However, the target genes that led to reduced fungal growth were limited and included effectors, cell wall modifiers, chitinases, and hexose transporters (Gebremichael et al., 2021). This is probably because gene silencing efficiencies do not always correlate with the extent of pathogen growth inhibition. For example, HIGS-based targeting of three *Fusarium graminis* ergosterol biosynthesis genes*, Cytochrome P450 (CYP) 51A*, *CYP51B*, and *CYP51C*, resulted in higher silencing of *CYP51A* (~85%) as compared to *FgCYP51B* and *FgCYP51C* (58%) but similar levels of fungal growth inhibition (Höfle et al., 2020). Similar observations were noted in *in-vitro* cultures of *F. culmorum*, where 90% silencing of *FcCYP51A* expression did not result in pathogen growth inhibition or morphological alterations, whereas 40% *FcCYP51B* silencing resulted in a 30% reduction in fungal growth and aberrant hyphal morphology (Koch et al., 2018). These findings suggest that *FgCYP51B* is essential for ergosterol biosynthesis, and thus fungal survival. Therefore, a successful RNAi outcome is determined by the essential nature of the target gene rather than its silencing efficiency, underscoring the importance of target gene selection.

Much effort is yet to be made in identifying potential candidate genes in various phytopathogenic fungi of agronomic importance. Apart from selecting target genes based on the knowledge of essential genes in other organisms, high throughput sequencing technologies and functional prediction tools may help identify a large number of candidate genes. Determining the expression level and subcellular localization of candidate genes will help filter down big data sets to the most crucial target gene(s), which would be simple to handle and analyze in-depth. Further, since the goal is to develop broad-spectrum resistance, the ability of these genes to serve as effective RNAi targets across phytopathogenic fungi should be investigated. It is also critical to find a core collection of genes that can influence pathogen survival at various stages of its life cycle. For instance, a comprehensive investigation into the role of *F. graminearum* RNAi machinery components in pathogenesis revealed that sexual ascosporogenesis is mediated primarily by *Dicer-like 1* (*DCL1*) and *ARGONAUTE 2* (*AGO2*), asexual conidia formation and germination by *DCL2* and *AGO1*, pathogenicity by *DCL1* and *AGO2*, and conidiation by RNA dependent RNA polymerases (RdRPs), RecQ helicase (QDE3) and AGO-interacting protein QIP (QDE2-interacting protein), indicating the essential role of these genes at different stages of the pathogen life cycle as well as their efficacy in RNAi-based *F. graminearum* control (Gaffar et al., 2019). Likewise, during infection, phytopathogenic fungi secrete virulence factors known as effectors at different infection stages for successful establishment on the host. Such genes with vital functions in promoting fungal pathogenesis should be given special attention because they are thought to be the most likely to inhibit growth when knocked down. Several studies have concentrated on mining effectors and characterizing their role in plant-pathogen interactions in various phytopathogenic fungi [e.g., (Sharma et al., 2019)]. However, not all effectors secreted by the fungi are equally effective in causing pathogenesis; therefore, pinpointing effectors that impact pathogen growth through SIGS is essential.

#### 3.1.2 Selection of target gene region for dsRNA production

RNAi efficiency can vary within specific regions of a gene. For example, adding artificial siRNAs (*asiR1245*, *asiR1362*, and *asiR1115*) targeting different regions of the transcription factor gene *MoAP1* to the culture media reduced *M. oryzae* growth and pathogenicity to different levels, with a*siR1115* being most effective against the rice blast fungus (Guo et al., 2019). Similarly, dsRNAs derived from different regions of the *Myosin 5* gene in *F. asiaticum* differed in their efficiency in reducing fungal growth under *in vitro* conditions as well as after spray application on wheat plants (Song et al., 2018). Gu et al. (2019) showed that the application of dsRNAs corresponding to four different but overlapping segments of an ~1.7 kb region of the *F. asiaticum β2-tubulin* gene resulted in varied gene silencing between 20%-90%. The dsRNA segment corresponding to nucleotides 917–1406 on the target *β2-tubulin* gene showed the highest level of silencing (90%) and a 92% reduction in lesion size compared to 20-30% silencing and 30% reduction in lesion size with the other segments. Likewise, dsRNAs targeted to four regions of *F. graminearum chitin synthase* (*Chs*), three regions of *Fg glucan synthase (Gls)*, and four regions of *Fg protein kinase (Pk)* genes showed transcript suppression ranging from 54%-60%, 58%-71%, and 27%-59%, respectively (Yang et al., 2021). These findings reveal that dsRNAs produced from different regions of the same gene have varied RNAi efficacies, the mechanism of which is not clearly understood in phytopathogenic fungi.

Research in insects has shown that the sequence of dsRNAs can affect their cleavage. For example, injection of dsRNAs into the Asian corn borer (*Ostrinia furnacalis*) and cotton bollworm (*Helicoverpa armigera*) tended to generate siRNAs specifically cut at GGU nucleotide sequences; dicing was diminished if these consensus sites were removed (Guan et al., 2018). On the contrary, dsRNAs were diced at AAG, GUG, and GUU locations in *Tribolium castaneum*. In paramecium, two classes of sRNAs, which are processed by DCL2, DCL3, and DCL5 regulate development. It has been shown that DCL2 has a stringent preference for 25 nt siRNA duplex and for the sequences 5′ U and 5′ AGA, whereas DCL3 prefers the sequence 5′ UNG. However, DCL5 prefers cleavage at 5′ UAG and 3′ CUAC/UN (Hoehener et al., 2018). In plants, preferential loading of small RNAs onto AGO proteins is determined by the presence of specific nucleotides at the 5′ of small RNA. For instance, AGO1 specifically recruits miRNAs that favor a 5′ terminal uridine, whereas AGO5 prefers cytosine (Mi et al., 2008). According to these studies, dsRNAs containing more consensus recognition sequences generate more siRNAs, resulting in high silencing efficiency. Another critical factor is the mRNA structure that can be accessed by a specific siRNA, with open secondary structures being more susceptible to silencing than closed secondary structures. Collectively, a combination of sequence biases associated with DCL processing, AGO loading, and mRNA structural accessibility to siRNA may contribute to variable RNAi efficiency in fungi.

#### 3.1.3 Variation in RNAi efficiency between genes

The effectiveness of RNAi can vary between genes within the same fungus when RNAi molecules are administered using the same method. SIGS-based targeting of *F. graminearum* RNAi machinery genes *AGO* and *DCL* resulted in varying silencing levels, with *FgDCL* (74%) suppressed more than *FgAGO* (40%). Silencing efficiencies can also vary between gene isoforms. For example, *FgAGO2* showed higher gene silencing (62%) than *FgAGO1* (50%), possibly due to the variation in the number of effective siRNAs generated in *FgAGO1* and *FgAGO2*. According to recent research conducted by Bayer laboratories, only 40% of the targeted gene products were silenced using externally applied siRNAs (Bennett et al., 2020). Additionally, the study found a weak relationship between target gene expression level and its down-regulation following siRNA administration, suggesting that highly expressed genes may be more effectively silenced. Therefore, it is important to carefully select RNAi targets, ensuring that they are expressed in tissues amenable to transcript suppression, in order to produce the desired phenotypes.

#### 3.1.4 Targeting multiple genes

The efficiency of gene silencing can be further enhanced by targeting multiple transcripts using a single dsRNA construct, adding repeats of the same dsRNA sequence, or mixing dsRNAs targeting multiple transcripts (Mumbanza et al., 2013; Koch et al., 2019). It has been demonstrated that simultaneous targeting of *B. cinerea DCL1* and *DCL2* transcripts using a double dsRNA construct (*DCL1/DCL2*) resulted in efficient gene silencing and a significant reduction in lesion size (90-100%) of *B. cinerea* infecting different hosts, including Arabidopsis, tomato, onion, grape, lettuce, rose, and strawberry (Wang et al., 2016). Koch et al. (2019) reported that transgenic expression (HIGS) of double dsRNA construct (*CYP-AC*, *CYP-BC*, *CYP-AB*) in barley and Arabidopsis engineered to target the *CYP51* genes (*CYPA*, *CYPB*, *CYPC*) of *F. graminearum* inhibited fungal growth more effectively than single dsRNA constructs (*CYP-A*, *CYP-B*, *CYP-C*), although both single and double dsRNA constructs reduced fungal infection. Likewise, spray application of combinations of two or three dsRNA constructs on detached wheat leaves targeting *F. graminearum Chs*, *Gls*, and *Pk* genes resulted in a greater reduction in lesion size than with individual constructs (Yang et al., 2021). Further, targeting *R. solani DCTN1*, *SAC1*, and *PG* transcripts with a single dsRNA construct for *DCTN1+SAC1* and *PG* alone resulted in a 58% and 43% reduction in fungal biomass and a significant reduction in disease symptoms in rice plants (Qiao et al., 2021). These studies suggest that simultaneous targeting of multiple transcripts leads to enhanced RNAi efficiency.

### 3.2 RNAi efficiency in different fungal species

A transcript that is more amenable to RNAi in one fungus may not be susceptible to RNAi in another, possibly due to differences in the sensitivity of the target species to RNAi. For instance, the application of dsRNA targeting the *β2-tubulin* gene of *F. asiaticum* infecting wheat resulted in crooked and exiguous mycelia and a 90% reduction in fungal biomass, whereas targeting the same gene in *C. truncatum* infecting soybean reduced fungal biomass by 54% (Gu et al., 2019). Notably, the RNA silencing pathways and their components viz., AGO, DCL, and RNA-dependent RNA polymerase (RdRP) appear to have diversified significantly in fungi (Nakayashiki and Nguyen, 2008). Some ascomycetes and basidiomycetes appear to lack some or all of the components (Nakayashiki et al., 2006; Li et al., 2010). Notably, *Ustilago maydis*, the fungus that causes maize smut, appears to have lost its entire RNA silencing machinery, making it resistant to plant-based RNAi control approaches (Laurie et al., 2008). *Zymoseptoria tritici*, in contrast, encodes the core components of the RNAi machinery but is still insensitive to dsRNA (Kettles et al., 2019). Therefore, prior to topical application of dsRNA (SIGS) for crop protection, it is crucial to establish through genome sequence analysis whether the target fungus contains the full complement of the RNAi machinery.

In plants, RdRPs are responsible for the onset of transitivity and the amplification/maintenance of RNAi (Willmann et al., 2011). Fungi also encode RdRPs (Wassenegger and Krczal, 2006); however, some species lack the components required for RdRP-based RNAi amplification, resulting in lower RNAi efficiencies in these species. For example, spraying dsRNAs targeting the *F. asiaticum Myo5* gene decreased fungal virulence in wheat only when a constant supply of dsRNA was available (Song et al., 2018). When the sRNAs were profiled by deep sequencing, *Myo5*-dsRNA-derived siRNAs were detected in plant cells but not in fungal cells, indicating that components involved in RdRP-based amplification of secondary siRNAs are not present in *F. asiaticum*. This strengthens the significance of fungal RdRPs in evoking the maximum RNAi response.

### 3.3 dsRNA-uptake efficiency in fungi

A fundamental prerequisite for RNAi-mediated plant defense against pathogens is effective dsRNA uptake by fungal cells. Various fungi have shown direct dsRNA and/or sRNA uptake, indicating the existence of uptake pathways [reviewed in (Cai et al., 2019; Wytinck et al., 2020; Šečić and Kogel, 2021)]. The mechanism for exogenous dsRNA uptake in *S. sclerotiorum* was recently identified as clathrin-mediated endocytosis (CME), a well-known and conserved eukaryotic pathway (Kaksonen and Roux, 2018; Wytinck et al., 2020). Labeling studies have revealed that certain fungi are adept at internalizing dsRNA while others are not. Using fluorescence imaging, labeled dsRNA uptake in *S. sclerotiorum*, especially at newly developing hyphal branches, was detected as early as two hours after co-incubation (McLoughlin et al., 2018). *Botrytis cinerea* spores grown on agar media and protoplasts isolated from liquid fungal cultures co-incubated with dsRNA after 12 or 20 hours, respectively, displayed fluorescein-labeled dsRNA or shorter sRNA fragments (Wang et al., 2016). Further, efficient dsRNA uptake was observed within 6 h in *Botrytis cinerea*, *Sclerotinia sclerotiorum*, *Rhizoctonia solani*, *Aspergillus niger*, and *Verticillium dahliae* (Qiao et al., 2021). In contrast, the same study showed that *Colletotrichum gloeosporioides* cannot internalize dsRNA, whereas limited uptake was observed in *Trichoderma virens*. These findings point to the need for further research into dsRNA uptake efficiencies to ensure that fungal pathogens can internalize dsRNA to trigger RNAi.

### 3.4 dsRNA/siRNA design

Owing to differences in potential siRNA generation capabilities among genic regions, identifying regions capable of producing maximum siRNAs is critical for RNAi efficacy. Several web-based tools are available to design effective siRNAs (Hajeri and Singh, 2009; Fakhr et al., 2016; Lück et al., 2019). They all use different algorithms and predict vulnerable siRNA generating regions differently. Although these tools are primarily employed to predict siRNAs in mammalian systems, they can also be used in other organisms, including fungi. Using a combination of web-based tools, Sharma et al. (2019) designed dsRNA ranging from 269-415 bp for targeting effectors and virulence genes in *E. pisi*. The siRNA vulnerable regions in the coding sequences of these target genes were identified using siRNA prediction tools: BLOCK-iT RNAi designer (https://rnaidesigner.lifetechnologies.com), Dharmacon (http://dharmacon.gelifesciences.com/design-center/), GenScript siRNA Target Finder (https://www.genscript.com/sslbin/app/rnai), RNAWizard (http://www.sirnawizard.com/design_advanced.php) and siRNA design (http://eu.idtdna.com/Scitools/Applications/RNAi/). The overlapping or consensus locations predicted to generate the maximum number of siRNAs by the different tools were selected for dsRNA synthesis. Also, regions close to the initiation codon (~100 bases) were avoided since they usually contain binding sites for different protein factors (Yuan et al., 2004). The dsRNA generated with these tools effectively suppressed the target *E. pisi* transcripts by 50-75%, confirming the feasibility of using these tools for fungal siRNA prediction.

Forster and Shuai (2020) used the Integrated DNA Technologies (IDT; Coralville, Iowa) software (https://www.idtdna.com/site/order/designtool/index/DSIRNA_CUSTOM) to design siRNAs targeting different regions of the *Chs6* gene specific to *Macrophomina phaseolina*. Two siRNAs designed to target the 3′ or 5′ ends of *MpCHS6* mRNA did not show any difference in their effectiveness in reducing *Mp* growth in *in-vitro* cultures. Werner et al. (2020) compared the RNAi efficacy of manually-designed dsRNAs (658-912 bp in length) with tool-designed dsRNAs (173-193 bp) against *F. graminearum AGO1/AGO2* and *DCL1/DCL2* genes, in which the respective sequences of manually- and tool-designed dsRNAs did not overlap. The manually-designed longer dsRNAs with the propensity to produce a larger number of effective siRNAs resulted in 4- to 10-fold higher silencing efficiencies than the shorter tool-designed dsRNAs, suggesting that the length of the dsRNA may be an important factor impacting RNAi efficacy in fungi and not necessarily the design method. However, Höfle et al. (2020) observed a modest decrease in SIGS-mediated *Fg* disease resistance with increasing dsRNA length, indicating that dsRNA size may interfere with the ability of *Fg* to internalize dsRNAs. To generate optimal sizes of dsRNA with maximum RNAi efficiency, precise identification of active siRNA generating regions by a tool-based approach is essential.

As an alternative to dsRNA, chemically synthesized unique siRNAs capable of producing maximum silencing efficiency can also be designed. The precise design of siRNAs is a critical step since only a few nucleotide changes within the sequence can alter its functionality (Harborth et al., 2003). As mentioned above, siRNAs designed with a combination of tools seem more efficient. While none of these tools can ensure 100% effectiveness, they all help maximize the likelihood of siRNA functionality. The following criteria are generally considered essential for siRNA design: (1) GC content of the sequence, (2) addition or deletion of specific nucleotides at particular positions, (3) thermodynamic parameters of siRNA duplexes and their targets, (4) structural requirements for their optimal action and (5) consideration of identical/similar sequences in their targets to avoid nonspecific activity (Hajeri and Singh, 2009; Fakhr et al., 2016). Further, studies that evaluated the performance of >100 siRNAs in *Drosophila* S2 cells, Chinese hamster, human HeLa, and HEK293 cells have suggested that higher A/U content at the 5’-end and higher G/C content at the 3’-end of the antisense strand result in greater silencing efficiencies (Reynolds et al., 2004; Ui-Tei et al., 2004; Jagla et al., 2005; Shabalina et al., 2006). Studies that considered these key design parameters yielded functional siRNAs with a mean percent silencing efficiency of 76-85%, while those chosen at random resulted in only 39% silencing efficiency (Reynolds et al., 2004). The effectiveness of siRNAs is also influenced by a number of other factors, including the distance between the target region and the transcription start site, accessibility of the target site, siRNA length, specificity check, base modifications to overcome off-target effects, secondary structures in the target site and siRNA, and the presence of asymmetry and energy valley within the siRNA (reviewed in Fakhr et al., 2016). This wealth of information is critical for effective siRNA design, and studies should focus on validating the factors mentioned above to see if similar siRNA parameters apply to phytopathogenic fungi for optimal RNAi efficiency.

### 3.5 dsRNA structure and dose

The structure of the dsRNA/siRNA is equally important in eliciting RNAi. In addition to the nucleotide composition of the siRNA duplex, base modifications and conjugates enhance gene silencing (Manoharan, 2004). Recent research has shown that small to medium-sized dsRNAs (21-60 bp) administered topically to plant cells require 2 bp 3’ overhangs for strong silencing activity (Bennett et al., 2020). siRNA conjugated with cholesterol, lithocholic acid, lauric acid or long alkyl branched chains (C32) at the 5’-end of the sense strand improved *in vitro* cell permeation in liver cells (Lorenz et al., 2004). Similarly, siRNA containing TAT peptide or TAT-derived oligocarbamate linked at the 3’-end of the antisense strand efficiently inhibited gene expression and localized to the perinucleus of HeLa cells without the need for a transfection agent (Chiu et al., 2004). It is essential to investigate whether similar base modifications and conjugations on dsRNA at the predicted siRNA loci can improve dsRNA uptake and RNAi efficacy in phytopathogenic fungi.

The other important aspect is the determination of the optimal dsRNA concentration that is required to enhance RNAi efficiency. In liquid cultures of *S. sclerotiorum*, the effects of different dsRNA doses (100, 200, 500, 1000 ng/mL) on target gene knockdown revealed that 200 ng/mL dsRNA was sufficient to achieve maximum knockdown among the targets (McLoughlin et al., 2018). In addition, it was shown that higher doses failed to produce a stronger silencing response once optimal knockdown was achieved. The effects of varied dsRNA concentrations (10^5^, 3.5x10^5^, 10^6^ ng/mL) on transgene silencing in Arabidopsis showed that the dsRNA concentration of 3.5x10^5^ ng/mL had the greatest effect on transgene-silencing efficiency (Dubrovina et al., 2019). It should be noted that the dsRNA dose optimized for a phytopathogenic fungus under *in vitro* conditions might not be effective in open-air conditions due to the impact of environmental conditions. To obtain consistent outcomes in the field, condition-specific dose optimization of dsRNA is required.

### 3.6 Off-target effects

Off-target effect (OTE) is referred to as the binding of siRNA/dsRNA sequences with unintended gene sequences of the target pathogen or another organism (Roberts et al., 2015) and is one of the major bottlenecks in RNAi applications. In order to reduce the risk of OTEs, RNA constructs for SIGS should be designed to target non-conserved sequences of the target gene. Further, the precise engineering of the siRNA/dsRNA duplex with all of the criteria that permit siRNA/dsRNA binding with high affinity to the target sequences favors mRNA degradation (reviewed in Hajeri and Singh, 2009; Fakhr et al., 2016; Schwartz et al., 2020). Despite these considerations, miRNA-like OTEs may occur. Strategies such as base modifications within the seed region of the siRNA guide strand, particularly at position 2 from the 5′ end, and siRNA pooling strategies, where different siRNAs are combined to lower the effective concentration of individual siRNAs in the pool, can reduce miRNA-like OTEs (reviewed in Neumeier and Meister, 2021). OTEs can also be resolved using genome-wide bioinformatics analyses that compare the designed siRNA/dsRNA sequence (partial or complete) and all known off-target mRNA sequences available for target and non-targeted organisms, including beneficial microbes. Alternatively, the NCBI’s BLAST tool can be used to evaluate both the sense and antisense strands of siRNAs, since either could potentially act as the guide RNA strand. Sequence homology < 78% with other genes, as few as 11 contiguous nucleotides, and ≤15 out of 19 nucleotides matching with the respective siRNA is believed to be tolerable (Jackson et al., 2003). Unfortunately, the NCBI BLASTn algorithm’s ability to accurately predict local alignments of short sequences limits its utility. As a result, dedicated bioinformatics software such as the open-access siRNA finder (si-FI) (https://github.com/snowformatics/siFi21) (Lück et al., 2019), ERNAi (https://www.dkfz.de/signaling/e-rnai3/), dsCheck (http://dscheck.rnai.jp/) and pssRNAit (https://plantgrn.noble.org/pssRNAit/) can be used to screen candidate dsRNA/siRNA sequences for complementarity with other genes.

Furthermore, exogenous RNAi application raises the possibility of unintentional epigenetic changes in plants as DCL3 is known to process dsRNA into 24-nt siRNAs, which are involved in RNA-directed DNA methylation (RdDM) of cognate DNA sequences (Zilberman et al., 2003; Chan et al., 2004). It was recently demonstrated that high-pressure spraying of dsRNA induces promoter methylation (Dalakouras and Ganopoulos, 2021). Spraying of fungal dsRNA that share a minimum of 30 bp sequence homology with plant DNA (gene or not), leads to DNA methylation of this locus in CG, CHG, and CHH context (Pélissier and Wassenegger, 2000). Importantly, CG and CHG methylation may be trans-generationally inherited even in the absence of the dsRNA trigger (Lindroth et al., 2001). Thus, SIGS OTEs can also be epigenetic in nature and trans-generationally maintained (Dalakouras and Papadopoulou, 2020). Careful design of dsRNA/siRNA and optimization of dsRNA spraying methods can help overcome these complications.

### 3.7 dsRNA delivery strategy

The effectiveness of RNAi depends on the RNAi molecules’ (dsRNA/siRNA) accessibility at the location of the target pathogen, which is determined by the delivery strategy. Studies comparing the SIGS versus HIGS mode of dsRNA delivery revealed that SIGS is more effective in silencing target gene expression than HIGS. SIGS-based delivery of single (*CYP-A*, *CYP-B*, *CYP-C*) or double (*CYP-AC*, *CYP-BC*, *CYP-AB*) dsRNA constructs targeting *CYP51* genes of *F. graminearum* reduced *Fg* growth by ~80% on barley. In comparison, HIGS-based administration of the same constructs reduced *Fg* growth by 39% and 9%, respectively (Koch et al., 2019). Under the conditions tested, SIGS was more effective than HIGS in reducing *Fg* growth, regardless of the dsRNA type (single versus double). A similar result was obtained when SIGS and HIGS were used to deliver dsRNAs of varying lengths to target the *FgCYP* genes on Arabidopsis (Höfle et al., 2020). SIGS-based dsRNA delivery was also superior to transgene-mediated expression in controlling *Phakopsora pachyrhizi* (Asian Soybean Rust) infection on soybean (Hu et al., 2020). The observed difference in RNAi efficiency may reflect the way in which the dsRNA is processed in SIGS versus HIGS. In HIGS, since the dsRNA is expressed in the plant cell, it may be primarily processed by plant DCL proteins (Meister and Tuschl, 2004). The resulting siRNAs may not have the optimal biochemical properties (size, modification, etc.) to exhibit efficient biological activity inside the fungal cell. On the contrary, in SIGS, a significant proportion of the dsRNA may be taken up directly by the fungus and processed by fungal DCLs into siRNAs that are optimal for fungal gene silencing (Dang et al., 2011).

The method of exogenous dsRNA/siRNA application on plants is also a critical aspect that defines SIGS efficiency (Dalakouras et al., 2016; Dalakouras et al., 2020; Nerva et al., 2020). Plant cells contain complex cellular structures such as the cuticle, cell wall, and plasma membrane, which operate as a physical barrier to exogenously applied molecules (Bennett et al., 2020). In addition, the dsRNA absorption capacities of various plant organs, such as leaves, petioles, buds, roots, stems, and seeds could also impact the effectiveness of exogenous dsRNAs (Das and Sherif, 2020). As a result, the introduction of exogenous RNA molecules into plant cells is considered an important factor for initiating RNAi. Generally, methods for exogenous dsRNA/siRNA application on plants include infiltration, injection, spreading, mechanical inoculation, root/seed soaking, and spraying (Dalakouras et al., 2018; Das and Sherif, 2020; Kiselev et al., 2021). Very recently, efficient delivery of DNA molecules into plant nuclei and chloroplast was achieved using cell-penetrating peptide (CPP) and chloroplast-targeting peptide-based delivery systems by foliar spraying (Thagun et al., 2022).

A study comparing different modes of dsRNA delivery such as high-pressure spraying of leaves, petiole adsorption, and postharvest spraying found that the reduction in *B. cinerea* disease symptoms varied with the dsRNA application method (Nerva et al., 2020). Postharvest spraying of grape bunches showed significantly reduced *Bc* growth followed by petiole adsorption and leaf spray. The greater reduction in *Bc* growth on grape bunches than in leaves despite using the same spray method suggests that grape bunches perceive dsRNA more than leaves. The mechanisms underpinning exogenously applied dsRNAs’ perception, recognition, and translocation into the plant cell are still unknown. According to a study conducted by (Niehl et al., 2016), exogenous dsRNA-induced PTI responses in Arabidopsis were found to be dependent on the co-receptor SOMATIC EMBRYOGENESIS RECEPTOR KINASE 1 (SERK1), which could act as a dsRNA receptor. The timing of dsRNA delivery also plays a crucial role in determining RNAi efficacy. For example, a greater reduction in disease symptoms was observed in rice leaves that were infected 24 h post spray (as opposed to 12 h) with *M. oryzae* spores mixed with dsRNA targeting *MoDES1*, a negative regulator of plant defense (Sarkar and Roy-Barman, 2021).

### 3.8 dsRNA stability

Studies in insects have shown that dsRNA stability can be enhanced by simultaneously delivering dsRNA targeting endonucleases to prevent target dsRNA degradation (Kaur et al., 2020). Further, dsRNAs synthesized to fold back on themselves like a paperclip (known as paperclip RNA or pcRNA) have been found to be more stable in the presence of endonucleases and suppress target transcripts in *Aedes aegypti* larvae even when the dsRNA uptake mechanism i.e., clathrin-mediated endocytosis was suppressed (Abbasi et al., 2020). This suggests that pcRNA is independent of clathrin-mediated endocytosis uptake in *A. aegypti* and may overcome concerns regarding dsRNA uptake-based mechanisms. Similar strategies could be adopted in phytopathogenic fungi, especially in those species where limited or no uptake of dsRNA is observed. Other promising methods, such as the coupling of a cationic oligosaccharide, oligodiaminogalactose 4mer (ODAGal4), and the incorporation of phosphorothioate linkages into siRNA, have been demonstrated to improve the biological and thermal stability of siRNAs *in vitro* (Irie et al., 2020). This strategy, when used in combination with siRNA pooling methods (reviewed in Neumeier and Meister, 2021), might help in the successful use of siRNA as crop protection tools by improving stability and reducing OTEs. Remarkably, none of the above-mentioned strategies has been employed for RNAi-based protection against phytopathogenic fungi.

## 4 Nanoparticles as carriers of RNAi molecules

One of the main limitations of the SIGS technology is the short window of protection conferred by topically applied dsRNAs. For effective RNAi, fungal-specific dsRNAs must be protected from degradation by RNases and released in a controlled manner into plant cells to provide prolonged RNAi-based protection against pathogens. Nanoparticles as carriers of dsRNA offer a cost-effective and potentially eco-friendly solution to improve the efficacy of the SIGS technology. The use of nanocarriers (NCs) for nucleic acid delivery in plant biotechnology is not new and dates back to the early 1980s. For example, liposomes were used to transport TMV RNA into tobacco BY-2 protoplasts to produce TMV particles (Nagata et al., 1981). Later, fluorescent conjugated polymer nanoparticles (CPNs) were used to deliver siRNAs targeting genes involved in the cellulose biosynthetic pathway into tobacco BY-2 protoplasts for gene silencing (Silva et al., 2010). These particles were non-cytotoxic but unable to cross the barrier of the plant cell wall and therefore not taken up by intact plant cells. Carbon nanotubes were among the first non-cytotoxic nanoparticles to be shown to cross the plant cell wall and membrane and deliver DNA cargo into intact tobacco BY-2 cells (Liu et al., 2020). It was not until 2017 that Mitter et al. (2017) demonstrated that dsRNAs targeting pathogen genes could be delivered to intact plant cells through topical spraying of layered double hydroxide (LDH) nanoparticle-loaded dsRNA onto intact plant leaves. They showed that loading of dsRNA onto LDH nanoparticles simultaneously protected and released dsRNA in a controlled fashion and provided longer-term protection against the cucumber mosaic virus (CMV) and pepper mild mottle virus (PMMoV) in cowpea than naked dsRNA alone. This was a pathbreaking discovery because it did not require any specific technique or chemical to load the dsRNA nor a complex delivery method such as transfection, gene gun bombardment, ultrasonic treatment, or magnetic force.

Nanoparticles possess several properties that make them excellent delivery vehicles. Firstly, the small size of nanoparticles (<100 nm in at least one dimension) provides a high surface area to volume ratio for effective cargo binding. Secondly, many nanoparticles are small enough to cross the plant cell wall and membrane barriers and can therefore efficiently deliver dsRNAs into plant cells. Thirdly, the ability to readily synthesize nanoparticles of different compositions, morphologies, sizes, and chemical properties makes them suitable for the transport of different types of biomolecules without toxic side effects.

### 4.1 Properties of an ideal nanocarrier

In addition to the above-mentioned features, a nanomaterial must possess the following properties to qualify as a dsRNA nanocarrier (NC). It should have a positively-charged core and/or surface to facilitate loading of negatively charged dsRNA. The zeta (ζ) potential of the NC in suspended form should be > +25 mV (Bao et al., 2016). It should have a low polydispersity index (PDI) ensuring uniform distribution in the solvent (Petrônio et al., 2022). The particle size, as estimated through TEM analysis, should be between 1-10 nm or 20-50 nm, to ensure translocation across the plant cell wall and membrane (Bao et al., 2016; Mitter et al., 2017; Zhang et al., 2019). The NC should be able to protect dsRNA from degradation thus extending the shelf-life of the biomolecule (Mitter et al., 2017). It should allow the persistent release of dsRNA on the surface or inside the plant cell, providing protection against the targeted pathogen for up to 30 days (Demirer et al., 2020). The NC should be water-soluble, reducing the requirement for organic solvents that may be toxic to plants. Lastly, but importantly, the NC should be biodegradable, biocompatible, and cost-effective to synthesize (Xu et al., 2006; Mosa and Youssef, 2021; Wang et al., 2021).

### 4.2 Nanocarriers tested for dsRNA-mediated RNAi against plant pathogens/pests

In this section, we present the NCs that have already been demonstrated to trigger RNAi in phytopathogens/pests through exogenous application of dsRNA-NC composites on plants. The physicochemical properties of these NCs and other characteristics that support their applicability as dsRNA nanotransporters are summarized in **Table 4** (I. Proven Nanocarriers).

**Table 4:** List of proven and probable nanocarriers for enhancing RNA-mediated gene silencing in phytopathogenic fungi/pests through exogenous application on plants.

Nanomaterial Type	Cargo	Pathogen/Pest [(target gene(s)]^1^	Plant species [target gene(s)]^2^	Delivery method	Physicochemical properties	Biocompatibility	Uptake in plant/pathogen	Reference
***I. Proven nanocarriers***								
MgAl layered double hydroxides (LDHs)	dsRNA	Cucumber mosaic virus (*CMV2b*), Pepper mild mottle virus (*PMMoVIR54*)	Cowpea, *Nicotiana tabacum*	Topical leaf spray	Size: 20-80 nm (TEM), 15-120 nm (Z-average) ζ potential: positive Polydispersity Index (PDI): 0.24	Degrades into biocompatible constituents (Mg^2+^, Al(OH)_3_ and NO_3_^−^, etc.)	Yes, demonstrated *via* Cy3-labeled RNA	Mitter et al., 2017
	dsRNA	Bean common mosaic virus (*Nib, CP*)	*N. benthamiana*, cowpea	Topical spray on detached leaves and/or seedlings	as in Mitter et al., 2017	–	–	Worrall et al., 2019
LDH	dsRNA	*Fusarium oxysporum (CYP51, Chs1, EF2)*	Tomato	Topical leaf spray, petiole adsorption, root dipping	Size: 50 nm (Z-average) ζ potential: positive PDI: 0.44	–	–	Mosa & Youssef, 2021
Chitosan nanoparticles (CNPs)	dsRNA	*Helicoverpa armigera* (pod borer) (*JHAMT, ACHE*)	Chickpea	Topical leaf spray	Size: 100 nm (TEM) ζ potential: +32 mV	Non-toxic in cell lines or non-target insects	Yes, demonstrated *via* Calcofluor-tagged CNPs	Kolge et al., 2021
Minicell (*Escherichia coli*-derived anucleated minicells)	dsRNA	*Botryotinia fuckeliana (grey mold) (Chs3a, Chs3b, DCL1, DCL2)*	*Strawberry*	Topical spray	Size: ~400 nm (Z-average)	–	–	Islam et al., 2021
Star Polycations (SPc)	dsRNA	*Aphis* *glycines* (aphid) (*TREH, ATPD, ATPE and CHS1*)	Soybean	Topical spray on seedlings, root soaking	Size: 100 nm (Z-average) ζ potential: +20.9 mV	–	Yes, demonstrated in root cells *via* Carboxy-X-Rhodamine (CXR)-labeled dsRNA	Li et al., 2019; Yan et al., 2020 (particle size)
	hpRNA	*Myzus persicae* (green peach aphid) (*ATPD*, *ATPG*)	Oilseed rape	Topical leaf spray	–	–	Yes, demonstrated in root cells *via GFP*-dsRNA	Ma et al., 2022
*II. Probable nanocarriers*								
DNA nanostructures	siRNA	*-*	Transgenic mGFP5 *N. benthamiana* (*GFP*)	Foliar infiltration	Size: 2 × 5 × 16 nm hairpin-tile monomer; 2 × 5 × 320 nm 10-unit nanostring; 2.4 nm for all edges of the tetrahedron (AFM)	No stress gene induction	Yes, demonstrated *via* Cy3-labeled DNA	Zhang et al., 2019
Carbon dots [Polyethyleneimine (PEI) functionalized]	siRNA	–	Transgenic GFP *N. benthamiana* and tomato (*GFP*, *CHLH*, *CHLI, magnesium chelatase*)	Topical leaf spray	Size: 2.7-3.9 nm (Z-average) ζ potential: +26.6 ± 1.6 mV PDI: 0.096-0.176	–	–	Schwartz et al., 2020
Gold nanocluster (PEI functionalized)	siRNA	–	Transgenic mGFP5 *N. benthamiana* (*GFP*), wildtype *N. benthamiana* (*ROQ1*)	Foliar infiltration	Size: ∼2 nm (TEM), 5−7 nm (Z-average) ζ potential: >+33 mV PDI: ~0.5	No stress gene induction	Yes, demonstrated *via* Cy3-labeled ssDNA oligo	Zhang et al., 2021
Single-walled carbon nanotube (SWNTs)	ssRNA (sense and antisense)	–	Transgenic mGFP5 *N. benthamiana* (*GFP, ROQ1*)	Foliar infiltration	Size: ssRNA-SWNT length: 776.6 nm, height: 1.567 nm (AFM)	No stress gene induction or tissue damage	Yes, demonstrated *via* Cy3-labeled RNA	Demirer et al., 2020
Cell penetrating peptide (CPP) N-terminal poly(lysine/histidine) domain-fused BP100 (KH9-BP100)	dsRNA	–	Transgenic *YFP A. thaliana (YFP, CHS)*	Foliar infiltration	Size: 100-300 nm (Z-average) ζ potential: >+30 mV	–	Yes, demonstrated *via* Cy3-labeled dsRNA	Numata et al., 2014
	siRNA	–	Transgenic *YFP Arabidopsis thaliana* (*YFP*)*;* Transgenic *GFP* tomato (*GFP*) Transgenic *N. tabacum* expressing *eGFP in* chloroplast	Topical leaf spray	Size: siGFPS1-KH9-BP100: ~264 nm (Z-average) PDI: ~0.7 Size: siGFPS1/KH9-OEP34-BP100: 107-560 nm (Z-average) PDI: 0.37-0.53	–	Yes, demonstrated *via* Cy3-labeled plasmid DNA	Thagun et al., 2022

^1^Pathogen gene names: CMV2b, 2b suppressor of the plant antiviral RNA silencing; PMMoVIR54, replicase gene; CYP51, cytochrome P450 lanosterolC-14a-demethylase; Chs, chitin synthase; EF2, elongation factor 2; Nib, nuclear inclusion b; CP, coat protein; JHAMT, juvenile hormone methyltransferase; ACHE, acetylcholine esterase; ATP, proton ATPase; TREH, trehalase; DCL, dicer-like; LUC, luciferase; IAP, inhibitor of apoptosis.

^2^Plant gene names: GFP, green fluorescent protein; ROQ1, Recognition of XopQ 1; CHL, magnesium chelatase; sfGN155, superfolder N-terminal GFP; sfGC155, superfolder C-terminal GFP; MxMT, 7-methylxanthine methyltransferase 1; YFP, yellow fluorescent protein; CHS, chalcone synthase.

#### 4.2.1 Layered double hydroxide (LDH) clay nanosheets

LDHs are inorganic nanomaterials composed of divalent and trivalent metal hydroxide brucite-like positively charged layers that are stabilized by H_2_O and interlayer anions like CO_3_^2-^, Cl^-^, NO_3_^-^, SO_4_^-^ (Xu et al., 2006). They are represented by the general formula: [(M^2^)_1 - x_(M^3^) _x_(OH)_2_]^x+^(A^m-^_x/m_).nH_2_O], where M^2^ and M^3^ are cations and A^m-^ is the anion in the hydrated interlayer (Kuang et al., 2010). The positively charged surface and the exchangeable interlayer anions allow these particles to load and release negatively charged molecules like RNA or DNA (Wu et al., 2018). By conjugating the negatively charged fluorescent dye FITC to LDH-lactate-nanosheets, Bao et al. (2016) showed that LDH particles of 0.5−2 nm thickness and 30–60 nm lateral diameters can be internalized into the cytosol of intact Arabidopsis seedlings.

Mitter et al. (2017) used these properties of LDH to deliver dsRNA targeting essential genes in the cucumber mosaic virus (CMV) and pepper mild mottle virus (PMMoV) for RNAi-mediated protection against these pathogens in cowpea and *Nicotiana tabacum*, respectively. Spray application of cowpea leaves with a 1:3 loading ratio of CMV2b-dsRNA-MgAl-LDH followed by CMV infection 1 or 5 days after treatment significantly reduced the formation of local necrotic lesions as compared with treatment with LDH alone. The study also demonstrated that dsRNA-LDH composites could provide long-term and systemic protection against CMV. Spray application of *N. tabacum* leaves with CMV2b-dsRNA-LDH followed by CMV challenge 20 days later in treated or untreated leaves conferred significantly higher protection against the virus than naked dsRNA alone. They further performed a comprehensive analysis to demonstrate the applicability of the LDH as an NC for RNAi-based protection under field conditions. The analysis showed that [1] dsRNA-LDH nanocomposites (BioClay) adhere to the leaf surface longer than dsRNA alone, [2] dsRNA delivered through LDH NCs internalize into plant cells and induce RNAi, [3] LDH NCs protect dsRNA from degradation, and [4] they breakdown into their constituent ions under normal environmental conditions, allowing for slow and sustained release of dsRNA. LDH nanosheets have also been tested as dsRNA NCs for protection against a fungal disease in tomato. MgAl-LDH nanosheets were loaded with dsRNAs targeting three essential genes [*CYP51*, *Chs1*, *elongation factor 2* (*EF2*)] sequences in a single fragment) in *F. oxysporum* and applied using different methods, including high-pressure foliar spray, petiole adsorption and root dipping (Mosa and Youssef, 2021). The study found that the spray method provided the maximum protection against the disease followed by petiole adsorption and root dipping indicating that the method of LDH-dsRNA delivery can affect the RNAi efficacy.

Factors that add to the scalability of LDHs include their low-cost synthesis (Xu et al., 2006) and biocompatibility (Gu et al., 2018). However, further studies on the biosafety of these NCs are required as LDH aggregates are toxic to mice (Yan et al., 2017) and green algae (Ding et al., 2018). The choice of the metal hydroxide used in the preparation of the LDH nanoparticles is also crucial. While they may not be toxic when present in the nanosheet form (Wu et al., 2022), their disintegration may release products that could contribute to the accumulation of toxic elements in certain environments. For example, Al(OH)_3_, a product of MgAl-LDH nanoparticle decomposition, may convert into phytotoxic Al species (Al^3+^) in acidic soils (Chandran et al., 2008; Bojórquez-Quintal et al., 2017).

#### 4.2.2 Chitosan nanoparticles (CNPs)

Chitosan is a biocompatible and biodegradable natural polysaccharide that can be easily modified to enhance its mechanical, chemical and biological properties for diverse applications (Negm et al., 2020). The positively charged amino groups in chitosan readily complex with the negatively charged phosphate groups from the RNA backbone. These unique properties make it an attractive NC for dsRNA delivery. The efficacy of CNPs as dsRNA delivery vehicles for RNAi-based pathogen control was recently demonstrated in chickpea against the insect pest *Helicoverpa armigera* (Kolge et al., 2021). The study showed that the ~ 100 nm-sized CNPs loaded and protected dsRNA from degradation by nucleases. The CNP-dsRNA composites were stable for up to 5 days on leaf surfaces, and internalization of dsRNA in the insect gut was shown *via* direct feeding with fluorescently-tagged CNPs; uptake into plant cells was not demonstrated. Topical spray application of CNP-dsRNA targeting genes with key functions in *H. armigera* growth and development significantly decreased expression of the specific genes and led to reduced larval feeding and pupa development on chickpea as compared to the control treatment, under lab and field conditions. Moreover, this effect was specific, as CNP-dsRNA targeting *H. armigera* genes did not negatively affect other Lepidopteran or Dipteran insects. Further, CNPs have been shown to be non-toxic to plants and human cell lines (Kolge et al., 2021; Petrônio et al., 2022), indicating that they are safe to use for broad applications.

#### 4.2.3 Minicells (Bacteria-derived anucleated nanoparticles)

Minicells are achromosomal nanoparticles derived from bacteria as a result of abnormal cell divisions (Farley et al., 2016). They possess all cell organelles like their parent cell but lack chromosomes. They are the only true biogenic nanomaterials that have been used as drug/siRNA delivery vehicles in human cancer therapeutics (2009; MacDiarmid et al., 2007). Minicells can easily encapsulate RNAi molecules such as siRNAs as they readily traverse the intact outer and inner membranes of these particles (MacDiarmid et al., 2009). Recently, (Islam et al., 2021) demonstrated the utility of *E. coli*-derived minicells in the synthesis and delivery of dsRNA targeting *Botryotinia fuckeliana* (grey mold) pathogenicity-related genes in strawberry. An *E. coli* mutant lacking RNase activity was transformed with DNA constructs to express dsRNAs targeting *B. fuckeliana* RNAi machinery (*DCL*) or cell wall integrity-related (*CHS*) genes and cultured in bioreactors to produce 100 mg/L minicell-encapsulated dsRNA. The dsRNA packaged in minicells were protected from nuclease degradation and able to specifically inhibit *B. fuckeliana* mycelial growth *in vitro*. Topical spray application of dsRNA-minicells on strawberries 1 h before *B. fuckeliana* inoculation completely inhibited fungal growth and disease development as compared to the minimal growth observed on naked dsRNA treatment. Further, complete protection against the disease was observed at 5 days postinoculation (dpi) when dsRNA-minicells (and not naked dsRNA) were applied 7 days prior to *B. fuckeliana* inoculation, demonstrating that the minicell-encapsulated dsRNAs can extend the protection window by up to 12 days.

Although minicells are relatively large nanoparticles (~400 nm), studies on human cell lines have shown that they can be efficiently internalized by human cells through the endosome-lysosome pathways, and once inside, degraded in the lysosome to release their siRNA cargo (MacDiarmid et al., 2009). The uptake of dsRNA-minicells by plant cells has not yet been demonstrated but they still hold great potential as NCs since, in addition to being able to deliver dsRNAs for gene silencing, they have the capacity to synthesize dsRNAs, which is one of the biggest hurdles in the scalability of nanoparticles for field applications.

#### 4.2.4 Star polycations (SPc)

Star polycations are cationic dendrimers consisting of a central core and positively charged peripheral arms that can form stable complexes with negatively charged nucleic acids (Xu et al., 2013). Among the many features that make SPc an appealing NC are its simple synthesis protocol, low cost of production, ability to be internalized into plant and insect cells, low cytotoxicity, and biodegradability (Xu et al., 2013; Li et al., 2019; Ma et al., 2022). SPc NCs have been used to transport dsRNA for RNAi-based management of insect pests such as the soybean aphid *Aphis glycines* (Yan et al., 2020) and the green peach aphid *Myzus persicae* (Ma et al., 2022). In both cases, topical spray application of dsRNA/hpRNA-SPc-detergent formulations targeting essential aphid genes on plants under laboratory (Yan et al., 2020) or greenhouse conditions (Ma et al., 2022) significantly reduced the expression of target genes and enhanced insect mortality rates. Ma et al. (2022) further improved the scalability of this system by developing a bacterial expression system for the mass production of hpRNA.

### 4.3 Prospective nanocarriers for RNAi in phytopathogenic fungi

In this section, we present NCs demonstrated to elicit RNAi of plant genes through exogenous application of dsRNA/siRNA-NC composites that can be potentially used for RNAi-based fungal control in plants. The physicochemical properties of these NCs and other characteristics that support their applicability as dsRNA nanotransporters are summarized in **Table 4** (II. Probable Nanocarriers).

#### 4.3.1 DNA nanostructures

DNA nanostructures are formed through the sequence-specific hybridization of single DNA strands and can be custom-designed to produce structures of various sizes and conformations. The ability to conjugate nucleic acid cargo to these nanoparticles *via* simple complementary base pairing makes them attractive for dsRNA delivery and gene silencing applications. This technology has been successfully implemented in animal systems for over a decade (Lee et al., 2012; Li et al., 2013). However, their applicability in plant systems was only recently explored. Zhang et al. (2019) showed that 1D hairpin-tile (HT) monomer and 3D tetrahedron nanostructures, assembled from four ssDNA oligonucleotides, could internalize into plant cells at a higher efficiency than a 1 D DNA nanostring when applied *via* foliar infiltration. The internalization depended on the nanostructure’s size, compactness, aspect ratio, and relative stiffness. The study reported that HT monomer and tetrahedron nanostructures with higher compactness and stiffness and at least one dimension below ~10 nm were more efficiently taken up by plant cells than the nanostring.

Zhang et al. (2019) further tested the ability of these DNA nanostructures to deliver siRNA molecules for RNAi-based gene silencing in transgenic mGFP5 expressing *N. benthamiana* leaves. The loading on DNA nanostructures protected siRNA from degradation and did not induce any stress response in plants. Foliar infiltration of siRNA-linked DNA nanostructures significantly reduced GFP expression in *N. benthamiana* leaves, demonstrating their RNAi-inducing ability. Notably, the extent of silencing correlated with the degree of nanostructure uptake and the location of siRNA attachment on the nanostructure. In terms of the duration of silencing, the exogenously applied siRNA-linked DNA nanostructures were able to suppress GFP expression until 7 days post infiltration, indicating that these NCs will have to be applied frequently to achieve long-term RNAi-based protection. However, the low cost of production of these DNA nanostructures may help offset expenses incurred due to their repeated administration.

#### 4.3.2 Carbon dots

Carbon dots are nano-sized carbon materials that have been utilized in diverse biomedical applications owing to their small size (<20 nm), biocompatibility, low toxicity, high water-solubility, and inherent fluorescence property (Liu et al., 2020). These nanoparticles can also be surface functionalized with amine groups for the attachment and delivery of negatively charged nucleic acids. Their application in plants as siRNA delivery vehicles for targeted gene silencing was recently reported as a breakthrough technology (Schwartz et al., 2020). The study showed that carbon dots synthesized from the cationic polymer polyethyleneimine (PEI) could load dsRNA and protect it from RNase degradation. To prove the efficacy of these nanoparticles as RNA delivery vehicles, 22 mer siRNAs were used instead of long dsRNAs. The study reported that topical spray application of siRNA-PEI carbon dots ranging in size from 2.7-3.9 nm was most efficient in silencing *GFP* in leaves of transgenic *GFP*-expressing *N. benthamiana* and tomato plants. Further, *GFP* silencing was also observed in younger unsprayed leaves 12 days after treatment, indicating that the silencing effect initiated by the siRNA could spread systemically in the plant. This nanoformulation was also effective in silencing endogenous genes in *N. benthamiana* with the silencing effect persisting up to 20 days post spray treatment. The ability of carbon dots to provide long-term systemic RNAi-based protection combined with their non-phytotoxic nature (Li et al., 2016) makes them well-suited for dsRNA delivery applications. However, further improvements in their synthesis and delivery method may be required to optimize mass production costs and gene silencing efficiencies for large-scale field applications.

#### 4.3.3 Single-walled carbon nanotubes (SWNTs)

SWNTs are one of the smallest (~1 nm) carbon-based cylindrical nanoparticles with diameters of 0.8-1.2 nm and lengths of 500-1000 nm (Demirer et al., 2020). Their large surface area to volume ratio allows them to load large quantities of cargo while their ultrasmall size and stiffness allow them to traverse the plant cell wall and membrane (Giraldo et al., 2014; Demirer et al., 2020). Since SWNTs are also able to cross the chloroplast lipid bilayer and become kinetically trapped within this organelle (Giraldo et al., 2014), they have been used as tools to deliver genes to chloroplasts of different plant species (Kwak et al., 2019).

Their utility in RNAi-based gene silencing in plants was recently demonstrated by Demirer et al. (2020). In this study, sense and antisense ssRNA targeting GFP were noncovalently adsorbed onto separate SWNTs and an equimolar mixture of sense and antisense loaded SWNTs was infiltrated into transgenic mGFP5-expressing *N. benthamiana* leaves. The study showed that the internalization of the ssRNA-SWNT complexes into plant cells was dependent on the length of the nanotubes with longer SWNTs (~780 nm) outperforming the shorter ones (~250 nm). *In vitro* experiments in which the ssRNA-SWNT complexes were incubated with plant cell lysate or water (control) demonstrated that the desorption of ssRNAs and subsequent hybridization into siRNA duplex occurs only in the intracellular milieu of the plant cell. In addition, the loading of ssRNA onto SWNTs protected them from nuclease degradation. The mixture of sense and antisense ssRNA-SWNT complexes could efficiently silence GFP in transgenic GFP-expressing *N. benthamiana* leaves 2 days after infiltration, but the effect was transient as levels of GFP returned to that of control leaves by 7 days after treatment. The ability of SWNTs to deliver ss molecules may be responsible for this transient effect as ssRNAs are generally more vulnerable to nuclease degradation than dsRNAs. Nevertheless, cytotoxicity tests indicate that these nanoparticles are highly biocompatible as they do not cause any tissue damage or stress gene induction (Demirer et al., 2020), making SWNTs suitable candidates for further exploration as siRNA carriers.

#### 4.3.4 Gold nanoclusters (AuNCs)

Surface functionalized gold nanoclusters ranging in size from ~2-100 nm have long been used for siRNA-based gene silencing in human cells (Kumar et al., 2019). Their small size, ease of synthesis, and ability to load and deliver RNA molecules make them attractive NCs for RNAi in plants. Their applicability in plant systems was recently demonstrated in a study by (Zhang et al., 2021), wherein PEI functionalized gold nanoclusters (PEI-AuNCs) were used to deliver 21-mer siRNA into wildtype or transgenic *GFP*-expressing *N. benthamiana* to silence an endogenous gene and *GFP*, respectively. The PEI-AuNCs could protect the siRNA from degradation and successfully silence the expression of the targeted plant genes with 80% efficiency. The ability of these NCs to internalize into mature plant cells was investigated by infiltrating different molar ratios of PEI-AuNC-ssDNA composites and various incubation times. The study reported that internalization of the infiltrated particles was maximal after 30 min-1 h post infiltration and when the PEI-AuNC to DNA ratio was greater than 50:1. Cytotoxicity assays revealed the absence of stress gene induction in PEI-AuNC-treated leaves 1 d after infiltration. However, longer-term cytotoxicity studies may be required to fully assess the biocompatibility of these NCs as a previous study reported leaf necrosis in tobacco plants 14 d after exposure to gold nanoparticles (Sabo-Attwood et al., 2012). Additionally, the ability of these NCs to provide systemic and sustained gene silencing remains to be ascertained.

#### 4.3.5 Cell penetrating peptide (CPPs)

CPPs are primarily short cationic peptides (~11-30 amino acids) that can permeate cell membranes of plant and animal cells (Chugh et al., 2010). Certain types of CPPs can directly bind nucleic acids through their positively charged residues whereas others can be fused to positively charged peptide polycations that are capable of forming ionic interactions with negatively charged nucleic acids (Numata et al., 2014). The low cytotoxicity of CPPs and the ability to bind cargo and traverse plant cell membranes have made them desirable carriers for dsRNA delivery applications. For example, Numata et al. (2014) fused a synthetic CPP named Bp100, previously shown to have bactericidal activity against phytopathogenic bacteria (Badosa et al., 2007), with a poly-lysine-histidine (KH)_9_ peptide to facilitate the attachment and delivery of dsRNA targeting the *YFP* gene. Once they confirmed that the fusion CPP could protect dsRNA from RNase degradation, they infiltrated the (KH)_9_-Bp100-dsRNA complex into the leaves of transgenic YFP-expressing Arabidopsis or poplar plants for dsRNA-mediated gene knockdown. They found that a CPP-dsRNA molar ratio of 2 was most efficient in silencing YFP and the silencing effect, which initiated between 9-12 h after infiltration, could partially sustain until 36 h. Further, the localization of fluorescently-labeled dsRNA near the nucleus indicated that the CPP had successfully delivered its cargo them into the cells. The same (KH)_9_-Bp100 CPP was later used to demonstrate the feasibility of simultaneously delivering multiple ssRNA biomolecules within the same plant cell (Thagun et al., 2020).

More recently, (KH)_9_-Bp100-siRNA complexes were successfully used for SIGS of *YFP* and *GFP* in transgenic YFP-expressing Arabidopsis and GFP-expressing tomato plants, respectively (Thagun et al., 2022). Spray application of (KH)_9_-Bp100 CPPs carrying a 27-mer YFP-targeting siRNA as cargo reduced YFP protein expression by 45% in transgenic YFP-expressing Arabidopsis plants 3 days after treatment. Similar results were observed after spray application of (KH)_9_-Bp100-siRNA (targeting *GFP*) in transgenic GFP-expressing tomato plants. However, the silencing efficiency observed with CPPs was lower than that observed with other GFP siRNA NCs, such as carbon dots, DNA nanostructures, and carbon tubes. One of the reasons behind the lower gene silencing efficiency of CPPs could be their lower internalization rates. The study found that the penetration efficiency of CPPs depends on the type of peptide that are attached to as well as the leaf architecture (e.g., stomatal density, trichome number, etc.) of the plant species they are sprayed on (Thagun et al., 2022). Therefore, extensive optimization may be required prior to utilizing CPPs as NCs of dsRNA/siRNA for large-scale applications. Interestingly, the same study showed that (KH)_9_-Bp100-siRNA complexes could also be targeted to the chloroplast for silencing chloroplast-expressed genes when coupled with a chloroplast transit peptide (CTP), indicating the viability of using CPPs for targeting organelle function.

## 5 Conclusions and future prospects

**Figure 2:**
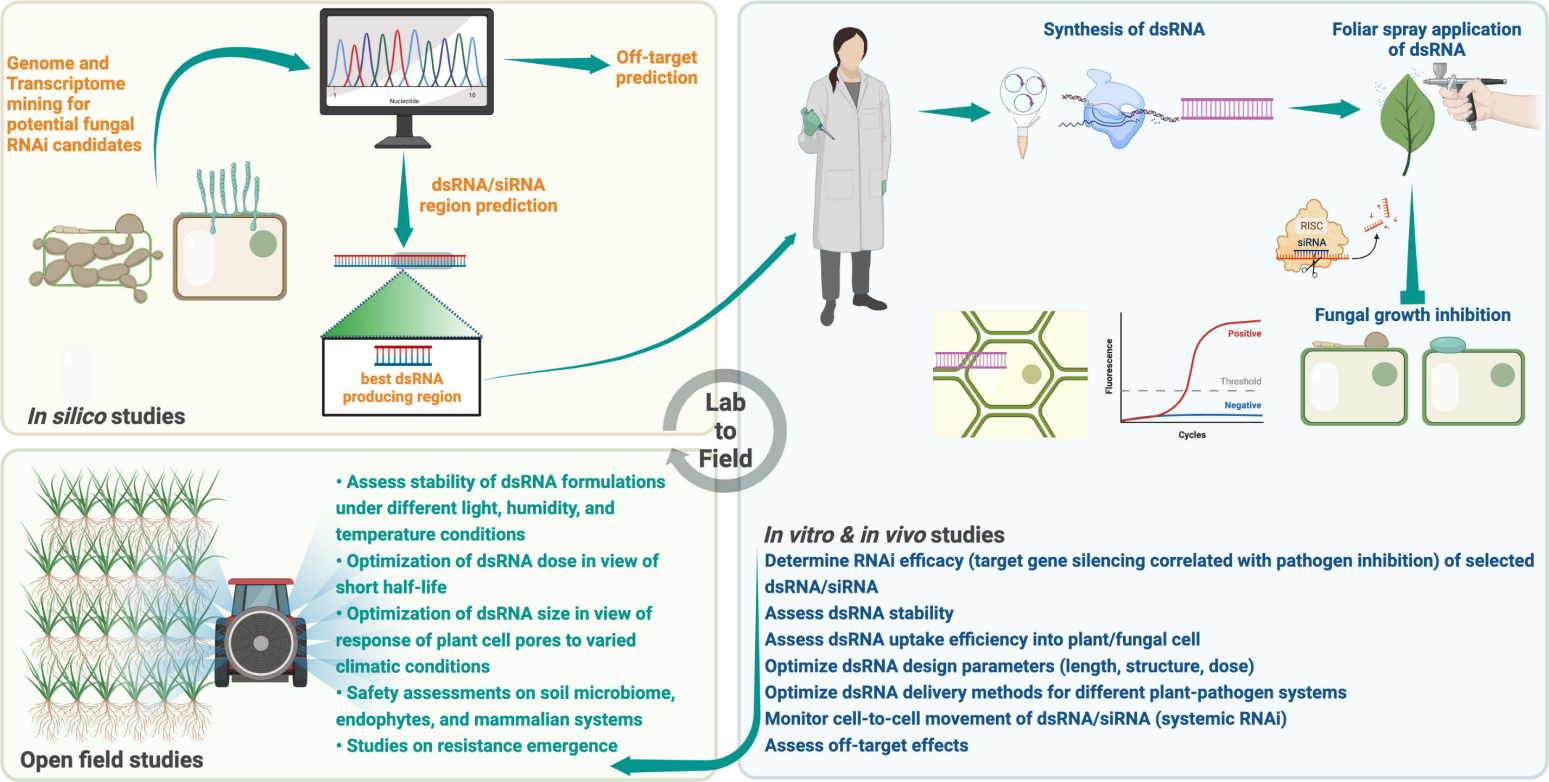
A checklist of experiments to consider for successful RNAi against phytopathogenic fungi and the development of innovative RNA-based fungicides for practical applications. Created with BioRender.com.

Owing to its simplicity of design, high specificity, and utility against a variety of pathogens, the application of target-specific dsRNA as an anti-fungal agent offers unprecedented potential as a new plant protection method (Islam and Sherif, 2020; Gebremichael et al., 2021; Rank and Koch, 2021). The success of RNAi-based fungal control relies on several factors as mentioned above, the significance of which is presented in **Figure 2** and **Table 3**. Despite a few limitations, the applicability of RNAi for plant protection against phytopathogenic fungi holds great promise.

Future efforts should focus on evaluating RNAi targets in a wide range of fungi. Also, it is essential to determine whether the dsRNA cleavage sites in plants and fungi are identical or unique. This will help researchers generate consensus/unique dsRNA that can be processed by the RNAi machinery of the host and fungus. Further, although efforts have been made to visualize the trafficking of small RNA between plant and fungal cells, the molecular mechanism behind this process is poorly understood and requires further research to improve RNAi efficacy in fungi that are refractory to RNAi.

For the practical application of dsRNA-based crop protection, it is also important to optimize the dsRNA size and dose under field conditions, as environmental factors such as light, temperature, and humidity can regulate plant cell wall openings and affect dsRNA stability. Future investigations into the possibility of hazardous and/or OTEs of the dsRNA/siRNA in the environment should provide crucial insights that will aid in the establishment of a regulatory framework for assessing potential biosafety issues.

The size of the NC has been a major factor in determining whether dsRNAs/siRNAs are internalized into plant cells. To traverse the plant cell wall and membrane that typically have a size exclusion limit of <20 nm, ultra-small nanoparticles such as DNA nanostructures, carbon dots, and SWNTs with large loading dsRNA/siRNA capacities have been developed. However, large NCs such as LDH and CNPs have been successfully used to confer RNAi-based protection against phytopathogens/pests, indicating that nanoparticle internalization may not be required for gene silencing. Future research may therefore focus on determining whether the size restriction for dsRNA delivery using NC poses a challenge to achieving the highest level of RNAi efficacy.

**Figure 3:**
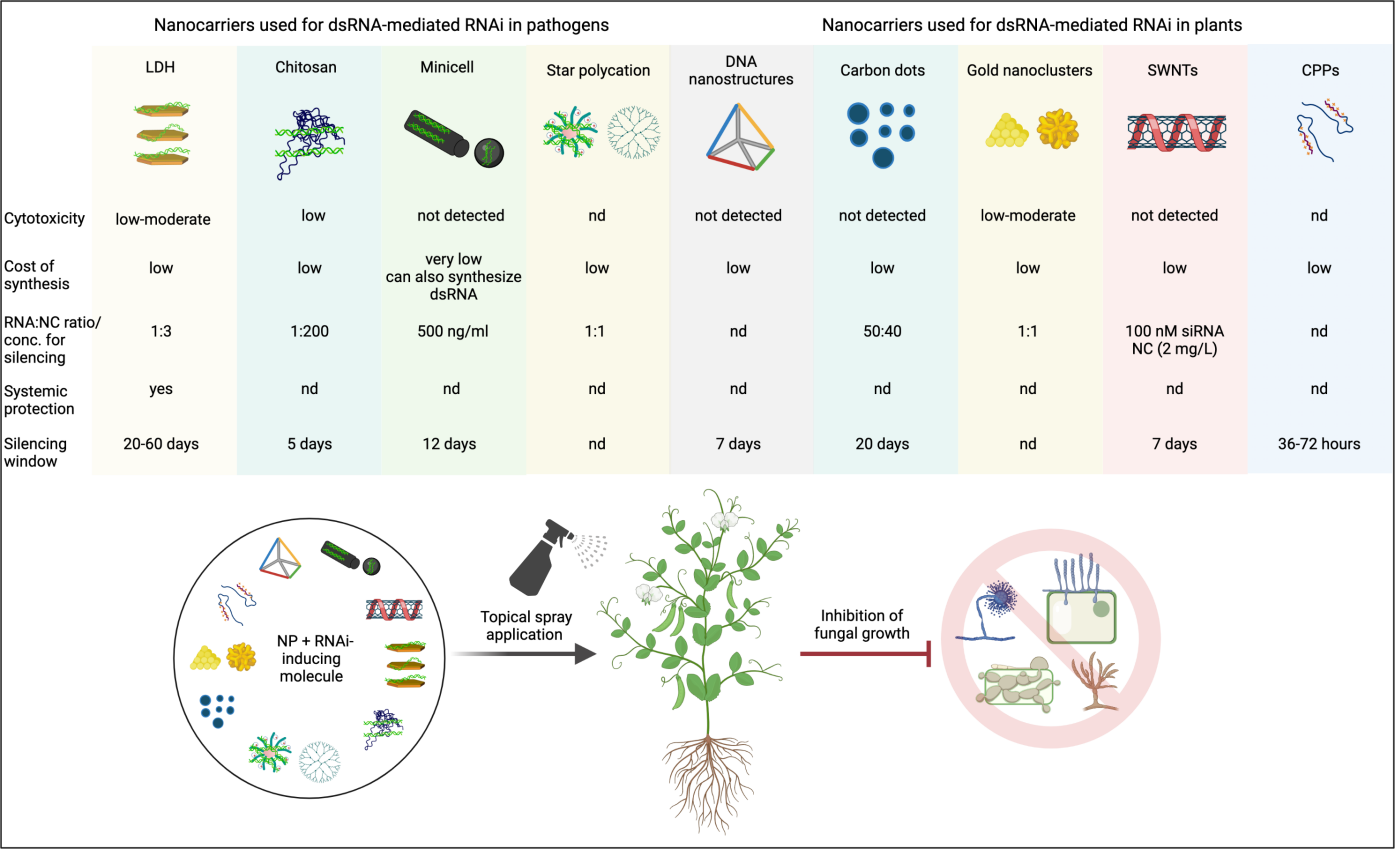
A summary of proven and potential nanocarriers and their key features that can be used to enhance the efficiency of RNAi-mediated control of phytopathogenic fungi in crops. NC, nanocarrier; NP, nanoparticle; LDH, layered double hydroxides; SWNTs, single walled carbon nanotubes; CPP, cell penetrating peptides. Created with BioRender.com.

Biocompatible and biodegradable NCs like LDH, chitosan, and carbon-based nanoparticles have already been tested as dsRNA/siRNA NCs in plants **(** **Figure 3**). However, a nanoparticle that potentially improves RNAi efficiency may be linked to higher toxicity in plants and animals and/or soil microflora. Therefore, studies on the safety assessment of nanoparticles should be conducted to mitigate the risks associated with them. Further, the potential of other promising NCs like mesoporous silica nanoparticles (MSNs), polyacrylic acid (PAA) coated hydroxyapatite (Elhaj Baddar et al., 2020), and poly-L-lysine (PLL) and polyphenol (-)-epigallocatechin gallate (EGCG) (Dhandapani et al., 2021) should be tested for SIGS in plants.

Undoubtedly, dsRNA-mediated crop protection *via* SIGS offers several advantages and has the potential to replace conventional fungicides in the near future. As evidenced by the studies discussed in this review, the proof-of-concept of RNAi in fungi is well established; however, the mass production of dsRNAs is a major bottleneck in the scalability of RNA-based crop protection programs. Utilizing microbial-based systems for dsRNA production or bacterial-based NCs (e.g., minicells) that can synthesize and deliver dsRNAs will help significantly lower the costs associated with RNAi-based fungal control in plants (Ahn et al., 2019; Islam et al., 2021; Hashiro and Yasueda, 2022). Indeed, the development of microbial-based dsRNA production systems has reduced the price of dsRNAs from ~ US$ 12,000/g in 2008, to US$ 1/g, with Greenlight’s GreenWorXTM system aiming to further lower the price of dsRNA synthesis to around US$ 0.5/g (reviewed in Guan et al., 2021). Based on the target species’ sensitivity to RNAi, systemic silencing capacity, and application method, it is estimated that ~2-10 g dsRNA would be required per hectare of arable land (reviewed in Das and Sherif, 2020). Therefore, at the dsRNA price of US$1/g, the cost of the SIGS technique could range between US$2-10 per hectare, making the practical application of SIGS technology for crop protection a reality today.

## Author contributions

PR, RA, and DC contributed to the conception of the manuscript, research, and writing. All authors contributed to the tables and figures. PR, RA, and DC wrote sections of the manuscript. RA and DC reviewed the work and wrote the final draft. All authors approved the submitted version.

## Funding

This work was funded by a Department of Biotechnology, Government of India grant (number BT/PR36172/NNT/28/1811/2021) to DC. PR and DS are supported by University Grants Commission Research Fellowships.

## Conflict of interest

The authors declare that the research was conducted in the absence of any commercial or financial relationships that could be construed as a potential conflict of interest.

## Publisher’s note

All claims expressed in this article are solely those of the authors and do not necessarily represent those of their affiliated organizations, or those of the publisher, the editors and the reviewers. Any product that may be evaluated in this article, or claim that may be made by its manufacturer, is not guaranteed or endorsed by the publisher.
